# One-Year Clinical Experience of Single-Port and Multi-Port Robotic Thyroid Surgery in a Single Institution

**DOI:** 10.3390/jcm13185486

**Published:** 2024-09-16

**Authors:** Sun Min Lee, Hilal Hwang, Myung Ho Shin, Jin Wook Yi

**Affiliations:** 1 Department of Surgery, Inha University Hospital & College of Medicine, Incheon 22332, Republic of Korea; ssunthy222@gmail.com (S.M.L.); hilalozer98@gmail.com (H.H.); tlsaudghg@gmail.com (M.H.S.); 2 Robot Surgery Center, Inha University Hospital, Incheon 22332, Republic of Korea

**Keywords:** thyroid, robotic surgical procedures, minimally invasive surgical procedures

## Abstract

**Background:** With the advent of da Vinci SP, surgical methods using da Vinci SP are becoming popular in thyroid surgery. The authors previously reported on a new surgical method called the single-port robotic areolar (SPRA) approach, which evolved from the previous bilateral axillary breast approach (BABA). This paper reports a comparative analysis of SPRA and BABA over one year. **Methods:** The data on SPRA and BABA thyroid surgery performed at the authors’ hospital from December 2022 to December 2023 were analyzed. **Results:** 111 SPRA and 159 BABA surgeries were performed. SPRA was performed overwhelmingly on women (1 man vs. 110 women), and the body mass index (BMI) was lower in SPRA patients (23.63 ± 3.49 vs. 25.71 ± 4.39, *p* < 0.001). The proportion of total thyroidectomy was significantly higher in BABA patients, and a modified radical neck dissection (MRND) was only performed using the BABA method. The time for flap formation before robot docking was significantly shorter in SPRA patients (12.08 ± 3.99 vs. 18.34 ± 5.84 min, *p* < 0.001). Postoperative drain amount was also significantly lower in SPRA patients (53.87 ± 35.45 vs. 81.74 ± 30.26 mL, *p* < 0.001). Hospital stay after surgery was significantly shorter with SPRA (3.04 ± 0.48 vs. 3.36 ± 0.73 days, *p* < 0.001). Thyroglobulin levels after a total thyroidectomy (0.06 ± 0.13 vs. 0.45 ± 0.78, *p* = 0.002) and stimulated Tg level before the RAI (1.03 ± 0.74 vs. 5.01 ± 13.63, *p* = 0.046) were significantly lower in the SPRA group. No significant differences were observed between the two groups according to the postoperative complications, including vocal cord palsy and hypoparathyroidism. **Conclusions:** Based on the authors’ experience, SPRA is a less invasive robot thyroid surgery method than BABA.

## 1. Introduction

Thyroid cancer is the most common cancer among all cancer types, with a rapidly increasing incidence in South Korea and worldwide [1]. Unlike other solid organ cancers, thyroid cancer occurs three to four times more often in women than in men and occurs in people in their 30s to 50s [2]. Most thyroid cancers have an excellent prognosis and a young age of onset. However, Asian people have a high incidence of keloids and hypertrophic scars, and due to cultural issues, it is very annoying for them to have surgical scars in places that are easily visible to others. Hence, various remote-access approaches have been developed to avoid leaving surgical scars on the front of the neck where the thyroid gland is located [3,4]. Previously, in the early 2000s, these remote-access surgical methods were performed primarily using laparoscopic surgical equipment [5,6]. On the other hand, the introduction of the da Vinci surgical robot in the mid-2000s has led to the replacement of laparoscope-based remote-access thyroid surgical methods with robot-assisted approaches [3,7,8]. The most representative robotic thyroid surgery methods include trans-axillary (TA), BABA(Bilateral Axillary Breast Approach), trans-oral robotic thyroidectomy (TORT), and robotic retro-auricular (RA) facelift thyroidectomy [9,10,11,12]. These robotic thyroid surgery methods have already been proven clinically in large-scale studies on surgical safety, complications, and oncological safety [13].

Before 2018, the da Vinci surgical robot only consisted of a multi-port four-arm-based system, including the da Vinci S, Si, and Xi. Thyroid surgery using this multi-port-based robotic surgical equipment requires a large subcutaneous space for the four robotic arms to move freely in the patient’s subcutaneous tissue [14]. Therefore, despite the various advantages of robotic surgery, it was difficult for existing robotic thyroid surgery to be recognized as minimally invasive surgery [3,15]. In 2018, the single-port-based da Vinci SP system was released in Korea, and trans-axillary approach thyroid surgery was performed using it for the first time [16]. Various centers have published their early experiences on thyroid surgery using the da Vinci SP system, which evolved from traditional multi-port-based robotic TA, BABA, and RA [17,18,19,20]. 

Robotic thyroid surgery in the authors’ hospital has used the BABA method since 2018 and reported considerable cumulative experience plus expansion of the surgical indication to a modified radical neck dissection (MRND) [21,22,23]. After installing the da Vinci SP in 2022, a new method called SPRA thyroidectomy, which uses breast access and has the same methodological advantages as conventional BABA surgery, was developed [18]. After successful initial reports of SPRA, two types of robot thyroid surgery were performed (SPRA and BABA) using the da Vinci SP and Xi system. As of 2024, no center using both methods simultaneously has been reported on. This paper reports the first comparative result of the clinical experience in SPRA and BABA surgery performed over one year.

## 2. Materials and Methods

The SPRA method began at the authors’ hospital in December 2022. This study retrospectively analyzed robotic thyroid surgery data, including electronic medical records and surgical video clips, for one year, from December 2022 to December 2023 [18]. Patients who underwent the BABA method were classified into the “BABA” group, and patients who received the SPRA method were classified into the “SPRA” group. The patients’ general information, such as age, gender, and BMI, was collected, and the results of the preoperative fine-needle aspiration cytology (FNAC) test and the location of the main tumor were collected from an electronic medical chart. The extent of thyroidectomy (lobectomy and total thyroidectomy and lymph node dissection–central lymph node dissection or less, modified radical neck dissection (MRND)) was collected from the surgical records. The surgical time was analyzed according to the extent of thyroid resection: lobectomy, total, and completion thyroidectomy. MRND was analyzed separately because it requires longer surgery times. 

**Figure 1:**
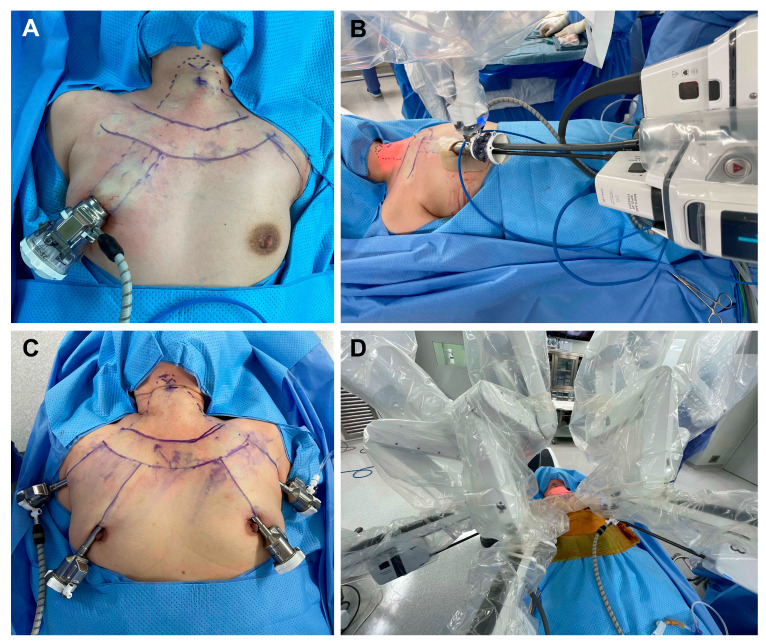
Robotic trocar positioning and robot docking in SPRA and BABA surgery. (**A**) Trocar placement for SPRA, (**B**) da Vinci SP docking in SPRA (**C**) Trocar placement for BABA, (**D**) da Vinci Xi docking in BABA.

A single endocrine surgeon with more than 1000 cases of robot surgery experience before starting SPRA performed all surgeries [22]. The steps of the SPRA and BABA procedures were the same as previously reported by the authors [14,18,21,23,24]. The position of the robot trocar and robot arm docking for SPRA and BABA are shown in Figure 1. The total operation time means from the start of a skin incision to the complete closure of the skin incision. The flap time was calculated from the insertion of the robot endoscope to the completion of the subcutaneous flap before the robot docking [14]. The console time was defined from when the surgeon started the robot wrist on the surgeon’s console to when the surgeon finished operating the console. The patients were discharged if their daily drainage amount was less than 50 mL. Vocal cord function after surgery was confirmed by vocal cord ultrasound in an outpatient clinic; this is because our outpatient clinic does not have a laryngoscope, only equipped ultrasound system [25]. Hypoparathyroidism after total thyroidectomy was defined as a case where the calcium levels decreased below 8.0 and calcium medication was required. The distinction between transient and permanent was based on the six months after surgery. 

Robotic thyroid surgery is performed without special differences in surgical indications from traditional open thyroid surgery in the authors’ hospital. An absolute contraindication of robot thyroid surgery is the clinical T4 stage, including direct invasion of the trachea, esophagus, vertebra, and common carotid artery, in a preoperative ultrasound and computed tomography (CT) scan. The SPRA method was recently developed. Hence, SPRA is generally recommended when the tumor size is 4 cm or less, whereas BABA can be performed on a tumor up to 10 cm in size. For central lymph node metastasis, SPRA or BABA are possible, except for tumors at a very deep, level 7 location. For lateral neck lymph node metastasis, BABA is generally recommended because of the extensive clinical experience associated with it, but SPRA was not recommended during this study period. 

Pathological characteristics were compared by dividing patients into three subgroups: papillary thyroid cancer (PTC) and other types of thyroid cancer; follicular thyroid cancer (FTC), Hürthle cell cancer, noninvasive follicular thyroid neoplasm with papillary-like nuclear features (NIFTP), medullary thyroid cancer (MTC), or benign thyroid tumor; and follicular adenoma, nodular hyperplasia, chronic lymphocytic thyroiditis, or Graves’ disease. Oncological completeness was analyzed using TSH-stimulated thyroglobulin (Tg) levels immediately before radioactive iodine (RAI) treatment in patients who received RAI after surgery. 

R version 4.4.1 was used for statistical analysis (www.R-project.org (accessed on 1 July 2024)). An unpaired t-test was applied to continuous variables, and Fisher’s exact test was used for cross-table analysis. The ethics of this study were approved by Institutional Review Board at the authors’ institution (IRB no: 2024-08-015). This research was registered at ClinicalTrials.gov (NCT06573268, date of registration: 25 August 2024).

## 3. Results

During the one-year study period, 270 patients received robot thyroid surgery at the authors’ institution. The BABA method was applied to 159 patients, and 111 patients received the SPRA method. Table 1 lists their clinical characteristics and surgery-related variables. The patients’ age was younger in the SPRA group, but the difference was not significant (42.86 ± 11.31 vs. 44.75 ± 11.49 years, *p* = 0.179). In the SPRA group, most patients were women; only one was male. In the BABA group, 50 and 109 patients were male and female, respectively. The body mass index (BMI) was significantly lower in the SPRA group (23.63 ± 3.49 vs. 25.71 ± 4.39, *p* < 0.001). The preoperative fine-needle aspiration cytology was mostly suspicious of PTC (Bethesda V) and PTC (Bethesda VI). The main tumor location (right and left side) was not different between the two groups (*p* = 0.784). Including bilateral tumors, the tumor location was significantly different (*p* = 0.009). For the extent of the thyroidectomy, a total thyroidectomy comprised a significantly higher proportion in the BABA group. A lymph node dissection in the central neck area (Level 6) was similarly performed in both groups, but an MRND was only performed in the BABA group. 

The total operation time was significantly shorter in the SPRA group when all patients were included in the analysis (101.94 ± 23.64 vs. 114.90 ± 32.83 min, *p* < 0.001). In subgroup analysis, the total operation time was similar in the two groups (98.75 ± 21.25 vs. 102.00 ± 18.39 min in lobectomy, *p* = 0.258, and 125.71 ± 26.23 vs. 125.70 ± 27.33 min, *p* = 0.998 in total thyroidectomy). The time for flap formation before robot docking was significantly shorter in the SPRA group (12.08 ± 3.99 vs. 18.34 ± 5.84 min, *p* < 0.001). The console time for the lobectomy and total thyroidectomy was similar in the two groups, 45.47 ± 12.93 vs. 43.72 ± 12.38 min (*p* = 0.339) in the lobectomy group and 68.00 ± 10.30 vs. 63.47 ± 16.38 min (*p* = 0.231) in the total thyroidectomy group. MRND required more console and total operation time (133.10 ± 25.55 min and 195.50 ± 36.55 min), but the significance could not be estimated because MRND was only performed in the BABA group. The estimated blood loss was similar in the two groups (20.81 ± 27.67 vs. 24.81 ± 48.545 mL, *p* = 0.392). Hospital stay days after surgery were significantly shorter in the SPRA group, in both lobectomy and total thyroidectomy patients, as listed in Table 1. Ten patients in SPRA and 50 patients in the BABA group received radioactive iodine therapy (RAI) after surgery.

Table 2 lists the postoperative surgical outcomes. There was no recurrent laryngeal nerve injury during surgery in both groups. Transient vocal cord palsy occurred in one patient in each group, but it was not permanent. Among the total thyroidectomy patients, transient hypoparathyroidism occurred in two patients in SPRA and 10 patients in the BABA group. Permanent hypoparathyroidism was not observed in either group. The laboratory test for PTH (Parathyroid hormone), calcium, and ionized calcium was similar in the two groups, as listed in Table 2. The amount of drainage in the postoperative first and second days was significantly lower in the SPRA group, regardless of lobectomy or total thyroidectomy (Table 2). The postoperative pain score was similar in the two groups. Two cases of seroma under the flap occurred in the BABA group. Postoperative hematoma occurred in one patient in each group, and they required hematoma evacuation surgically but experienced no prolonged complications.

Table 3 lists the pathology findings of the enrolled patients. The final pathologic diagnosis was mostly PTC. Other cancers included five follicular thyroid cancers (FTCs), one Hurthle cell cancer, five noninvasive follicular thyroid neoplasms with papillary-like nuclear features (NIFTPs), and one medullary thyroid cancer (MTC). Tumor size was significantly larger in the BABA group, in total (0.80 ± 0.48 vs. 1.38 ± 1.21 cm, *p* < 0.001), and in the PTC (0.69± 0.25 vs. 1.14± 0.89 cm, *p* < 0.001) subgroup. The number of retrieved central lymph nodes was similar in the two groups (4.84 ± 4.02 vs. 4.56 ± 4.06, *p* = 0.60). The numbers of metastatic lymph nodes and the proportions of BRAFV600E and TERT promotor mutations were similar.

Table 4 lists the surgical completeness in the total thyroidectomy and completion thyroidectomy patients who received RAI after surgery. The postoperative three-month serum thyroglobulin level was significantly lower in the SPRA group (0.06 ± 0.13 vs. 0.45 ± 0.78 ng/mL, *p* = 0.002). RAI dose and TSH level before RAI were similar in the two groups. TSH-stimulated thyroglobulin levels before RAI administration were significantly lower in the SPRA group (1.03 ± 0.74 vs. 5.01 ± 13.63 ng/mL, *p* = 0.046). This section may be divided by subheadings. This should provide a concise and precise description of the experimental results and their interpretation, as well as the experimental conclusions that can be drawn.

## 4. Discussion

The development of medical technology is progressing rapidly in modern society. Just as the paradigm changed from long-standing open surgery to laparoscopic surgery in the 1990s and 2000s, laparoscopic surgery has shifted rapidly to robotic surgery since the 2010s [26]. The advantages of the da Vinci surgical robot system are natural 3-D HD vision without using special image-converting glasses, mimicking of the surgeon’s hand movement using Endo-wrist technology, scaled and filtered movement from the surgeon’s motion that leads to precise and safe surgery, and many types of surgical devices with da Vinci specialized specifications for various areas of surgery. In addition, compared to laparoscopic surgery, the surgeon can perform the surgery ergonomically while sitting with his/her arms supported; the surgeon’s fatigue is much less than in laparoscopic surgery [27]. With the recent launch of the single-port-based da Vinci SP system, minimal invasiveness has been added to the advantages of robotic surgery. It expands robotic surgery applications through less traumatic surgery with precise surgery for patients [28]. 

In thyroid surgery, remote-access surgical methods have been actively attempted because of the unique epidemiologic characteristics, such as young age onset and frequently occurring in females [2]. These remote-access thyroid surgery methods, like other areas of surgery, have been attempted using laparoscopic equipment. Nevertheless, prior laparoscopic-based thyroid surgery methods have gradually moved to robot-based methods because of the spread of robotic surgical devices [3,4]. Among many remote-access thyroid surgery methods, this study focused on BABA surgery because the author mainly performed robotic BABA thyroid surgery, and newly developed SPRA thyroid surgery is also a method evolved from BABA surgery [18,21,22,23]. The advantages of BABA surgery over other surgical methods are as follows. First, any surgeon familiar with open surgery can perform BABA surgery without changing the surgical method because the surgical process is performed, as in open transcervical surgery, by looking down from the top. Second, the view of both thyroid glands is the same; there is no problem with performing a total thyroidectomy using the same surgical view without changing the instruments. Third, BABA does not require additional equipment, such as a retractor; fewer complications are encountered because of the traction of the arm after surgery. Lastly, there is sufficient evidence for applying the BABA method to cancer surgery because cervical lymph node dissection, including MRND, is possible, and oncological safety after surgery has been proven [10,15,21,22]. On the other hand, the most important disadvantage of BABA surgery is that a wide range of flaps are required, from both axillae through the anterior chest to the neck. Paresthesia or pain occurs below the flap area, and the probability of seroma increases after BABA surgery [14,29,30]. Therefore, although the BABA surgical method is considered remote-access surgery, calling it minimally invasive surgery is difficult [3,15]. 

The authors attempted to overcome the disadvantages while maintaining the advantages of BABA surgery. This paper reports for the first time on a new surgical method called SPRA, in which the da Vinci SP robot is docked only through a right areolar incision [18]. The SPRA method can be called “minimally invasive” because the area of the subcutaneous flap has been reduced by more than 50% compared to BABA surgery. The comparative result in Table 1 shows that the flap time was significantly shorter in the SPRA group than in the BABA group (12.08 ± 3.99 min vs. 18.34 ± 5.84 min, respectively). The daily drainage amount was significantly lower in the SPRA group, as described in Table 2. This result may be because the SPRA group has a smaller subcutaneous flap area than the BABA flap. The hospital stay days after surgery were significantly shorter in the SPRA group (3.04 ± 0.48 vs. 3.36 ± 0.73 days, respectively) because discharge was permitted only when the daily drainage volume was reduced to less than 50 mL. Nevertheless, the pain level felt by patients after surgery was similar in the two groups, as shown in Table 2. Seroma under the flap only occurred in two cases in the BABA group, which was resolved after repeated aspiration in the outpatient clinic. Vocal cord palsy and hypoparathyroidism, which are the most important postoperative outcomes in thyroid surgery, were very low in both groups, and no significant differences were found, as shown in Table 2.

In the pathologic evaluation, the diagnosis of most patients was PTC, as shown in Table 3. Tumor size was significantly smaller in the SPRA group because SPRA surgery was developed more recently than BABA, and patients with relatively small tumors were mainly prescribed SPRA. Unlike tumor size difference, extrathyroidal extension was not different between the two groups. The number of retrieved central lymph nodes and metastatic central lymph nodes was similar with the two methods. During the study period, MRND was only performed using the BABA method. On the other hand, SPRA MRND was initiated in 2024, and this paper reports on the initial result of SPRA MRND in the near future. In particular, the postoperative thyroglobulin level after total thyroidectomy was significantly lower in the SPRA group: both TSH unstimulated (0.06 ± 0.13 vs. 0.45 ± 0.78, *p* = 0.002, respectively) and TSH stimulated (1.03 ± 0.74 vs. 5.01 ± 13.63, *p* = 0.046, respectively), as listed in Table 4. The proportion of stimulated Tg under 1.0 was similar in the two groups. With these results, the SPRA method can allow for a more complete elimination of thyroid tissue in a total thyroidectomy. On the other hand, the SPRA group mainly selected patients with small tumors, so selection bias may have occurred. More clinical experience with a larger number of patients will be needed to support surgical completeness in SPRA. 

Compared to other existing surgical methods using the da Vinci SP, the main advantage of the SPRA method is that the thyroid glands can be easily accessed under the same robot placement. TA or RA allows for easy access to the thyroid gland on the same side as robot docking, but it is challenging to perform surgery on the thyroid gland on the side opposite the one where the robot is docked [3]. In addition, compared to TA surgery, which requires a separate retractor to maintain the subcutaneous flap, SPRA is less invasive to the patient because it does not require a retractor [16,20]. In the case of TORT, which has been widely attempted recently, there is a risk of unwanted complications, such as mental nerve injury, oral commissure injury, and surgical site infection [11]. On the other hand, these concerns are not a problem with the SPRA method. In addition, only the multi-port robotic surgical device using the TORT method has been reported, and TORT using the da Vinci SP has not yet been reported. Compared to conventional BABA, SPRA is a much less invasive surgery because it requires a small subcutaneous flap. Therefore, considering these results, the SPRA method is a new surgical method that is the most minimally invasive, allows for complete bilateral thyroidectomy, and is safe for surgical and oncological outcomes. The cosmetic results are much better than those of open surgery.

The limitations of this study are as follows. Patients who were likely to be easy to operate on would have been assigned to the SPRA group by the surgeon because SPRA had just begun two years ago. This acts as a selection bias. Therefore, future studies should conduct propensity score matching after more cases are accumulated or perform randomized controlled trials to overcome selection bias. In addition, the surgical outcomes (vocal cord palsy, hypoparathyroidism, and other complications) tended to be very good in both groups in the present study. This is probably because the surgeon in this study was an expert with clinical experience in more than 1000 cases of robotic thyroid surgery before he developed the SPRA method. Research will also be needed on the experiences of new surgeons when performing the SPRA thyroidectomy, and research on the learning curve is needed. For the surgical indications, there was only one male patient in the SPRA group because the size of male areolae is smaller than in women, so it is difficult to make a 3 cm incision around the peri-areolar area in male patients. Additionally, sometimes the location of the thyroid gland is deeper than in women, so it is difficult to approach the thyroid gland using a limited single-port system. Lastly, experience with MRND was not included because SPRA is in its early stages in this study. As of 2024, MRND is gradually being implemented using SPRA, which is also shown to be sufficiently feasible by SPRA method. This will be reported elsewhere in the near future. Additional consideration of robotic surgery is the cost. Robotic surgery is known to be 3–5 times more expensive than general open or laparoscopic surgery. As the economics of South Korea have been growing very fast, most patients have private insurance in addition to national insurance, so the economic problem of robotic surgery is gradually being diluted in South Korea. However, because financial support for robotic surgery may vary by country due to political and economic issues, we must consider this issue when approaching robot surgery. 

## 5. Conclusions

Summarizing the authors’ one-year comparative study on SPRA and BABA surgery, SPRA is a good surgical method that maintains the advantages of BABA surgery because it is minimally invasive. Large-scale comparative studies with propensity score matching will be conducted as the number of patients increases. In addition, comparative studies with conventional open transcervical thyroid surgery will be conducted. Furthermore, more experience will be needed to expand the indications for surgery, such as MRND, Graves’ disease, and huge goiter.

## Figures and Tables

**Table 1:** Clinical and surgery-related variables.

Variable	Total	SPRA (*n* = 111)	BABA (*n* = 159)	*p*-Value
Age (years, mean ± SD, (range))	43.97 ± 11.43 (17–76)	42.86 ± 11.31 (20–67)	44.75 ± 11.49 (17–76)	0.179
Gender				
Male	51	1	50	<0.001
Female	219	110	109	
BMI (body mass index, kg/m^2^)	24.85 ± 14.16	23.63 ± 3.49	25.71 ± 4.39	<0.001
Fine-needle aspiration cytology (Bethesda category)				<0.001
Papillary thyroid cancer (VI)	113	47	66	
Suspicious of papillary thyroid cancer (V)	98	48	50	
Follicular neoplasm or suspicion for a follicular neoplasm (IV)	17	9	8	
Atypia of undermined significance (III)	11	5	6	
Benign (II)	20	1	19	
Nondiagnostic/inadequate (I)	2	0	1	
For completion thyroidectomy	10	1	9	
Tumor location				0.009
Right	138	63	75	
Left	88	38	50	
Bilateral	41	8	33	
Isthmus	3	2	1	
Thyroidectomy extent				<0.001
Lobectomy	193	96	97	
Total thyroidectomy	67	14	53	
Completion thyroidectomy	10	1	9	
Lymph node dissection				0.006
Central neck dissection or less	260	111	149	
Lateral neck dissection (MRND *)	10	0	10	
Total operation time (minutes, mean ± SD)	109.57 ± 30.04	101.94 ± 23.64	114.90 ± 32.83	<0.001
Lobectomy	100.38 ± 19.88	98.75 ± 21.25	102.00 ± 18.39	0.258
Total thyroidectomy	125.70 ± 26.83	125.71 ± 26.23	125.70 ± 27.33	0.998
Total thyroidectomy plus MRND	195.50 ± 36.55		195.50 ± 36.55	
Completion	109 32 ± 64	75.00	112.78 ± 32.22	
Flap time (minutes, mean ± SD)	15.88 ± 6.02	12.08 ± 3.99	18.34 ± 5.84	<0.001
Console time (minutes, mean ± SD)				
Console time for lobectomy	44.59 ± 12.65	45.47 ± 12.93	43.72 ± 12.38	0.339
Console time for total thyroidectomy	64.58 ± 15.16	68.00 ± 10.30	63.47 ± 16.38	0.231
Console time for total thyroidectomy plus MRND		NA	133.10 ± 25.55	NA
Completion thyroidectomy	48.5 ± 16.68	36.00	49.89 ± 17.07	NA
Estimated blood loss (mL, mean ± SD)	23.17 ± 41.24	20.81 ± 27.67	24.81 ± 48.545	0.392
Hospital stay days after surgery (days, mean ± SD)	3.23 ± 0.66	3.04 ± 0.48	3.36 ± 0.73	<0.001
Hospital stay days after lobectomy	3.13 ± 0.52	3.05 ± 0.49	3.22 ± 0.54	0.029
Hospital stay days after total thyroidectomy	3.31 ± 0.78	2.93 ± 0.47	3.44 ± 0.83	0.006
Hospital stay days after total thyroidectomy plus MRND			4.00 ± 0.82	NA
Hospital stay days after completion	3.8 ± 1.23	3	3.89 ± 1.27	
Radioactive iodine therapy (numbers)	60	10	50	<0.001

* MRND: modified radical neck dissection.

**Table 2:** Postoperative outcomes.

Variable	SPRA (*n* = 111)	BABA (*n* = 159)	*p*-Value
Vocal cord palsy			1.000
Transient (<6 months)	1	1	
Permanent (>6 months)	0	0	
Hypoparathyroidism *			
Transient (<6 months)	3	8	0.795
Permanent (>6 months)	0	2	
PTH ^†^ (pg/mL) < 2 weeks *	8.35 ± 4.95	9.08 ± 7.42	0.703
PTH ^†^ (pg/mL) > 6 months *	15.51 ± 7.40	15.72 ± 8.51	0.938
Calcium < 2 weeks *	8.18 ± 0.41	8.23 ± 0.52	0.738
Calcium > 6 months *	9.10 ± 0.34	9.30 ± 0.47	0.128
Ionized calcium < 2 weeks *	1.00 ± 0.09	1.01 ± 0.09	0.871
Ionized calcium > 6 months *	1.19 ± 0.06	1.19 ± 0.98	0.974
Drain amount for postoperative first day, lobectomy (mL)	53.87 ± 35.45	81.74 ± 30.26	<0.001
Drain amount for postoperative first day, total thyroidectomy (mL)	55.20 ± 23.54	105.10 ± 50.62	<0.001
Drain amount for postoperative second day, lobectomy (mL)	30.92 ± 21.04	38.47 ± 25.00	0.024
Drain amount for postoperative second day, total thyroidectomy (mL)	32.50 ± 17.96	50.11 ± 42.22	0.040
VAS ^‡^ for postoperative 1st day	2.75 ± 0.49	2.71 ± 0.55	0.565
VAS ^‡^ for postoperative 2nd day	2.34 ± 0.63	2.35 ± 0.63	0.835
Other complications			0.932
Seroma	0	2	
Flap eczema	0	1	
Wound problem	1	1	
Hematoma	1	1	

* Only evaluated from total thyroidectomy and completion thyroidectomy patients. ^†^ PTH: parathyroid hormone. ^‡^ VAS: visual analogue scale for pain, 0 to 10.

**Table 3:** Pathological findings.

Variable	SPRA (*n* = 111)	BABA (*n* = 159)	*p*-Value
Pathologic diagnosis			
PTC *	100	132	0.248
Other cancers ^†^	4	8	
Benign ^‡^	7	19	
Tumor size (cm)	0.80 ± 0.48	1.38 ± 1.21	<0.001
PTC *	0.69 ± 0.25	1.14 ± 0.89	<0.001
Other cancers ^†^	1.85 ± 0.31	2.84 ± 1.52	0.115
Benign ^‡^	1.92 ± 0.97	3.08 ± 1.97	0.115
Extrathyroidal extension ^¶^			
Absent	72	92	0.128
Present	34	49	
Lymph nodes, retrieved			
Central node dissection (CND)	4.84 ± 4.02	4.56 ± 4.06	0.60
Modified radical neck dissection (MRND)		30.80 ± 10.30	
Lymph nodes, metastatic			
Central node dissection (CND)	1.23 ± 2.20	1.21 ± 1.85	0.928
Modified radical neck dissection (MRND)		10.7 ± 5.034	
*BRAF*^V600E^ mutation			0.368
Absent	13	23	
Present	91	113	
*TERT* promotor mutation			0.237
Absent	103	130	
Present	1	5	

* Papillary thyroid cancers. ^†^ Follicular thyroid cancer, Hürthle cell cancer, noninvasive follicular thyroid neoplasm with papillary-like nuclear features (NIFTP), MTC. ^‡^ Follicular adenoma, nodular hyperplasia, chronic lymphocytic thyroiditis, Graves’ disease. ^¶^ Including microscopic and gross extrathyroidal extension.

**Table 4:** Surgical completeness in total thyroidectomy and completion thyroidectomy patients.

Variable	SPRA (n = 10)	BABA (n = 50)	*p*-Value
Postoperative three months Tg * (mean ± SD, ng/mL)	0.06 ± 0.13	0.45 ± 0.78	0.002
1st RAI ^†^ dose (mean ± SD, mCi)	80 ± 25.82	99 ± 35.7	0.064
TSH level before RAI ^†^ (mean ± SD, μIU/mL)	129.42 ± 47.03	109.68 ± 40.79	0.24
Stimulated Tg level before RAI ^†^ (mean ± SD, ng/mL)	1.03 ± 0.74	5.01 ± 13.63	0.046
<1 (number of patients)	5	18	0.485
>1 (number of patients)	5	32	

* Tg: thyroglobulin. ^†^ RAI: radioactive iodine.

## Data Availability

The data that support these findings are available upon request from the corresponding author.
